# The genome sequence of 18-spot Ladybird, *Myrrha octodecimguttata *(Linnaeus, 1758)

**DOI:** 10.12688/wellcomeopenres.23455.1

**Published:** 2025-01-14

**Authors:** Maxwell V.L. Barclay, Talay Namintraporn, Michael F. Geiser, Natural History Museum Genome Acquisition Lab, Darwin Tree of Life Barcoding collective, Wellcome Sanger Institute Tree of Life Management, Samples and Laboratory team, Wellcome Sanger Institute Scientific Operations: Sequencing Operations, Wellcome Sanger Institute Tree of Life Core Informatics team, Tree of Life Core Informatics collective, Darwin Tree of Life Consortium

**Affiliations:** 1 Natural History Museum, London, England, UK

**Keywords:** Myrrha octodecimguttata, 18-spot Ladybird, genome sequence, chromosomal, Coleoptera

## Abstract

We present a genome assembly from an individual male specimen of *Myrrha octodecimguttata* (18-spot Ladybird; Arthropoda; Insecta; Coleoptera; Coccinellidae). The genome sequence has a total length of 769.90 megabases. Most of the assembly (84.54%) is scaffolded into 10 chromosomal pseudomolecules, including the X sex chromosome. The mitochondrial genome has also been assembled and is 20.61 kilobases in length. Gene annotation of this assembly on Ensembl identified 22,423 protein-coding genes.

## Species taxonomy

Eukaryota; Opisthokonta; Metazoa; Eumetazoa; Bilateria; Protostomia; Ecdysozoa; Panarthropoda; Arthropoda; Mandibulata; Pancrustacea; Hexapoda; Insecta; Dicondylia; Pterygota; Neoptera; Endopterygota; Coleoptera; Polyphaga; Cucujiformia; Coccinelloidea; Coccinellidae; Coccinellinae; Coccinellini; *Myrrha*; *Myrrha octodecimguttata* (Linnaeus, 1758) (NCBI:txid703265).

## Background

*Myrrha octodecimguttata* (Linnaeus, 1758) is a widespread species found across the Palaearctic region including North Africa and Iberia to Syria and the Levant, spanning through to the Russian far east, Kazakhstan and Mongolia ( Kovář, 2007). In the United Kingdom and Ireland this species is widespread and locally common in appropriate habitats ( Roy *et al.*, 2013). *M. octodecimguttata* can be found in coniferous woodland and is closely associated with Scots Pine *Pinus sylvestris* ( Majerus, 1994; Roy *et al.*, 2013). Both the adults and larvae are commonly found in the canopy layer of mature pine trees, where they can be the most abundant species of Coccinellidae ( Czechowska, 1995). They primarily feed on aphids in the pine trees in which they live, but unlike other conifer specialists, 18-spot ladybirds will not change their host tree in search of food when resources become scarce. Instead, they either move to another pine tree nearby, or remain until aphid numbers increase ( Roy *et al.*, 2013). 18-spot ladybirds will also feed on adelgids and pollen ( Majerus, 1994).

The genus name *Myrrha* was coined by Etienne Mulsant (1797–1880) who gave names from Greek mythology to many ladybird genera. The mythological ‘Myrrha’ was the mother of Adonis, and was transformed into a tree, so perhaps an appropriate name for a very strongly arboreal coccinellid.

*M. octodecimguttata* is a distinctive ladybird species, characterised by its orange colouration and 18 cream-coloured spots. This colouration may be required for both camouflage and to warn predators, as this species possesses alkaloids which can be used as a chemical defence ( Roy *et al.*, 2013). In Britain, the only similar species is *Calvia quattuordecimguttata* (Linnaeus, 1758), which has fewer (14) spots arranged in a different pattern, and is larger and less strongly convex. Most specimens of *M. octodecimguttata* have the basal cream-coloured spots around the scutellum arranged in a characteristic shape of an ‘M’ for ‘ *Myrrha*’.

Adults of *M. octodecimguttata* will emerge from their overwintering sites in April and newly emerged adults have been observed from June ( Majerus, 1994; Roy *et al.*, 2013). Adults overwinter in the crowns of pine trees, often as aggregations of several individuals. These aggregations have also been found to include other species of Coccinellidae such as the larch ladybird, *Aphidecta obliterata* (Linnaeus, 1758) ( Roy *et al.*, 2013).

**Figure 1.**
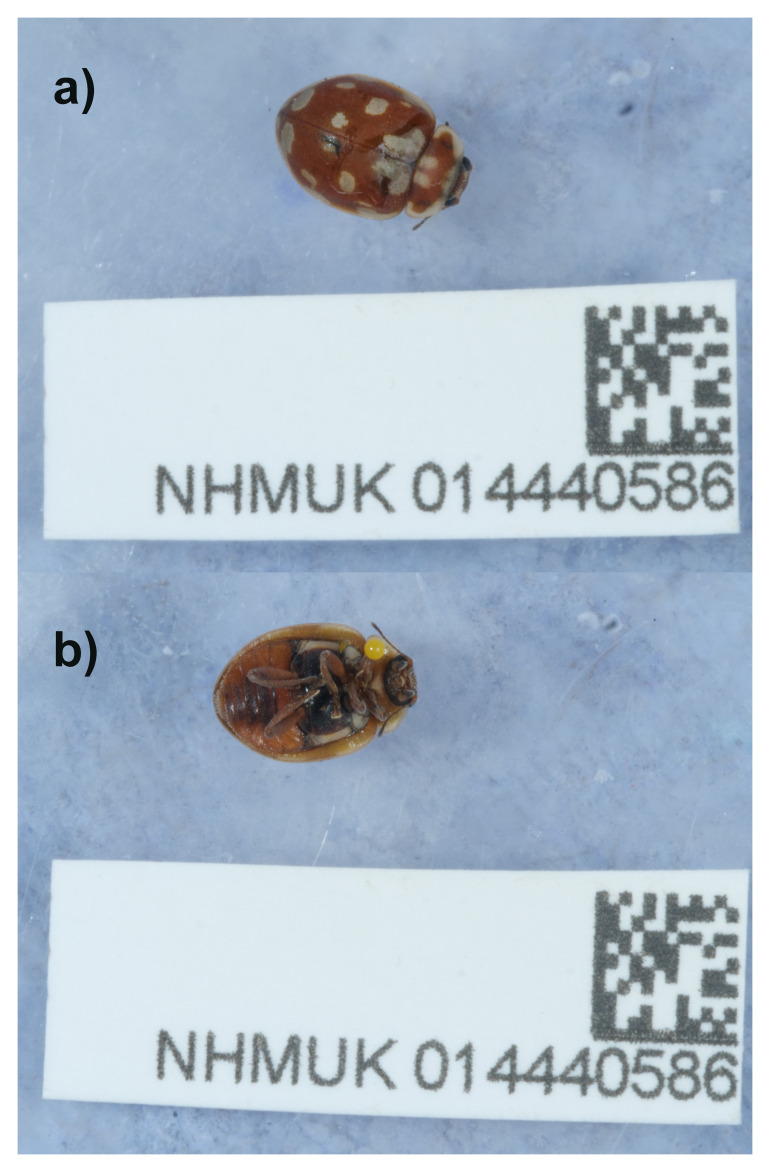
Photograph of the *Myrrha octodecimguttata* (icMyrOcto1) specimen used for genome sequencing.

We present a chromosome-level genome sequence for *Myrrha octodecimguttata* ( Figure 1), based on a male specimen from Brompton Cemetery, England, United Kingdom. Brompton Cemetery in the Royal Borough of Kensington and Chelsea, London, is a Grade 1 Listed site managed by the Royal Parks; it is well known as a haven for wildlife, and includes trees dating back to the 1830s ( Friends of Brompton Cemetery, 2024). A selection of mature ornamental pines, including several non-native species of *Pinus*, support large populations of aphids of the genera *Cinara* and *Eulachnus* (Hemiptera: Aphididae) which in turn provide food for *M. octodecimguttata* which is extremely abundant in the Cemetery.

## Genome sequence report

The genome of *Myrrha octodecimguttata* ( Figure 1) was sequenced using Pacific Biosciences single-molecule HiFi long reads, generating a total of 24.23 Gb (gigabases) from 2.23 million reads, providing an estimated 29-fold coverage. Primary assembly contigs were scaffolded with chromosome conformation Hi-C data, which produced 113.37 Gb from 750.77 million reads. Specimen and sequencing details are summarised in Table 1.

**Table 1.** Specimen and sequencing data for *Myrrha octodecimguttata*.

Project information			
**Study title**	Myrrha octodecimguttata		
**Umbrella BioProject**	PRJEB61846		
**Species**	*Myrrha octodecimguttata*		
**BioSample**	SAMEA112221799		
**NCBI taxonomy ID**	703265		
Specimen information			
**Technology**	**ToLID**	**BioSample accession**	**Organism part**
**PacBio long read sequencing**	icMyrOcto1	SAMEA112221880	Whole organism
**Hi-C sequencing**	icMyrOcto1	SAMEA112221880	Whole organism
Sequencing information			
**Platform**	**Run accession**	**Read count**	**Base count (Gb)**
**Hi-C Illumina NovaSeq 6000**	ERR11439631	7.51e+08	113.37
**PacBio Sequel IIe**	ERR11413975	2.23e+06	24.23

Assembly errors were corrected by manual curation, including 19 missing joins or mis-joins and 5 haplotypic duplications. This reduced the scaffold number by 1.39% and increased the scaffold N50 by 35.71%. The final assembly has a total length of 769.90 Mb in 1,066 sequence scaffolds, with 86 gaps, and a scaffold N50 of 72.7 Mb ( Table 2).

**Table 2.** Genome assembly data for *Myrrha octodecimguttata*, icMyrOcto1.1.

Genome assembly		
Assembly name	icMyrOcto1.1	
Assembly accession	GCA_958510865.1	
*Accession of alternate haplotype*	*GCA_958510795.1*	
Span (Mb)	769.90	
Number of contigs	1,153	
Number of scaffolds	1,066	
Longest scaffold (Mb)	89.46	
Assembly metrics *		*Benchmark*
Contig N50 length (Mb)	9.7	*≥ 1 Mb*
Scaffold N50 length (Mb)	72.7	*= chromosome N50*
Consensus quality (QV)	64.0	*≥ 40*
*k*-mer completeness	primary 76.21%; alternate: 61.53%; combined: 98.08%	*≥ 95%*
BUSCO **	C:97.8%[S:96.0%,D:1.8%], F:0.8%,M:1.4%,n:2,124	*S > 90%, D < 5%*
Percentage of assembly mapped to chromosomes	84.54%	*≥ 90%*
Sex chromosomes	X	*localised homologous pairs*
Organelles	Mitochondrial genome: 20.61 kb	*complete single alleles*
Genome annotation of assembly GCA_958510865.1 at Ensembl		
Number of protein-coding genes	22,423	
Number of gene transcripts	22,665	

* Assembly metric benchmarks are adapted from Rhie *et al.* (2021) and the Earth BioGenome Project Report on Assembly Standards September 2024. ** BUSCO scores based on the endopterygota_odb10 BUSCO set using version 5.3.2. C = complete [S = single copy, D = duplicated], F = fragmented, M = missing, n = number of orthologues in comparison. A full set of BUSCO scores is available at https://blobtoolkit.genomehubs.org/view/icMyrOcto1_1/dataset/icMyrOcto1_1/busco.

**Figure 2.**
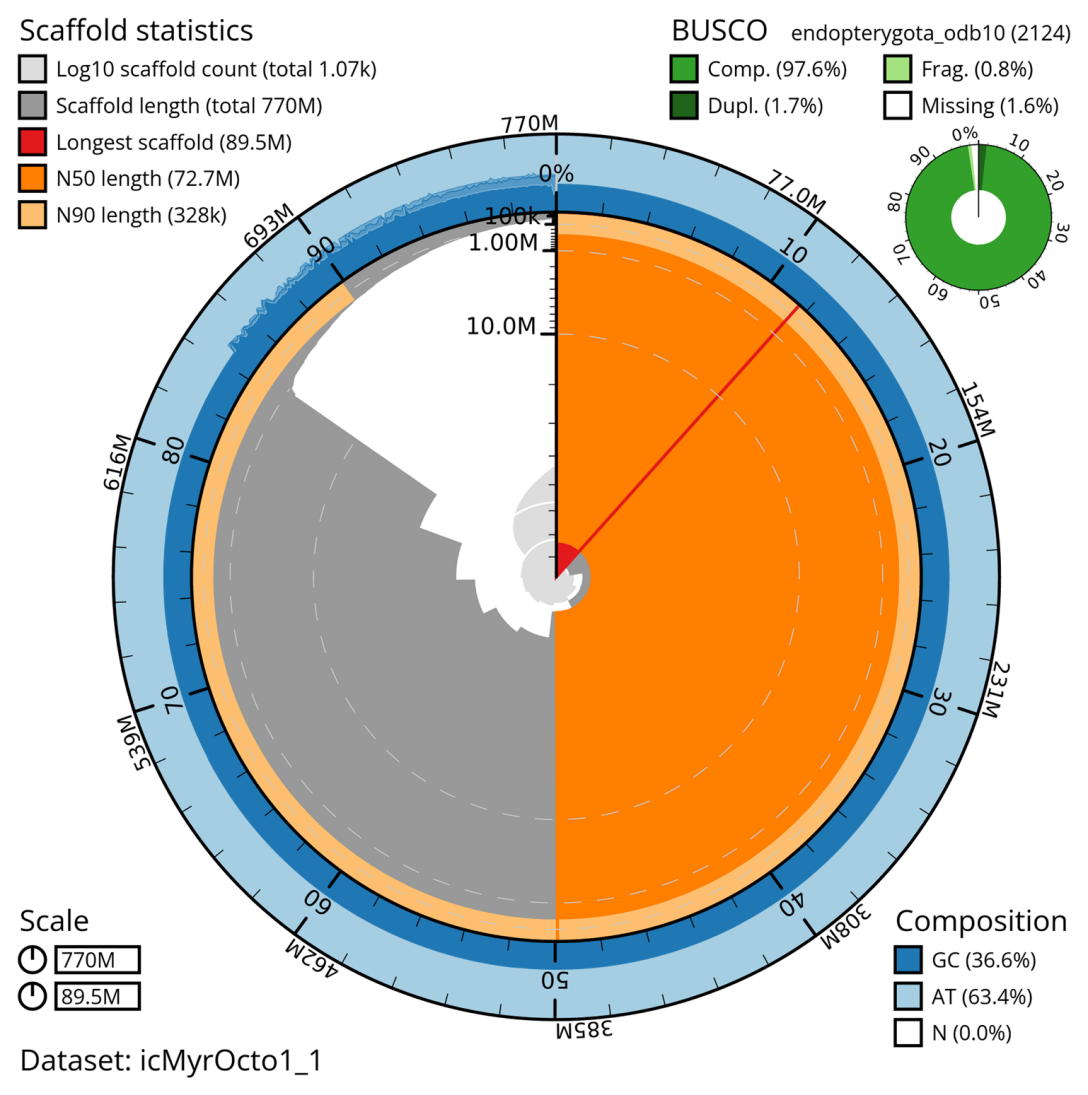
Genome assembly of *Myrrha octodecimguttata*, icMyrOcto1.1: metrics. The BlobToolKit snail plot provides an overview of assembly metrics and BUSCO gene completeness. The circumference represents the length of the whole genome sequence, and the main plot is divided into 1,000 equal-sized bins around the circumference. The outermost blue tracks display the distribution of GC, AT, and N percentages across the bins. Scaffolds are arranged clockwise from longest to shortest and are depicted in dark grey. The longest scaffold is indicated by the red arc, and the deeper orange and pale orange arcs represent the N50 and N90 lengths. A light grey spiral at the centre shows the cumulative scaffold count on a logarithmic scale. A summary of complete, fragmented, duplicated, and missing BUSCO genes in the endopterygota_odb10 set is presented at the top right. An interactive version of this figure is available at https://blobtoolkit.genomehubs.org/view/icMyrOcto1_1/dataset/icMyrOcto1_1/snail.

**Figure 3.**
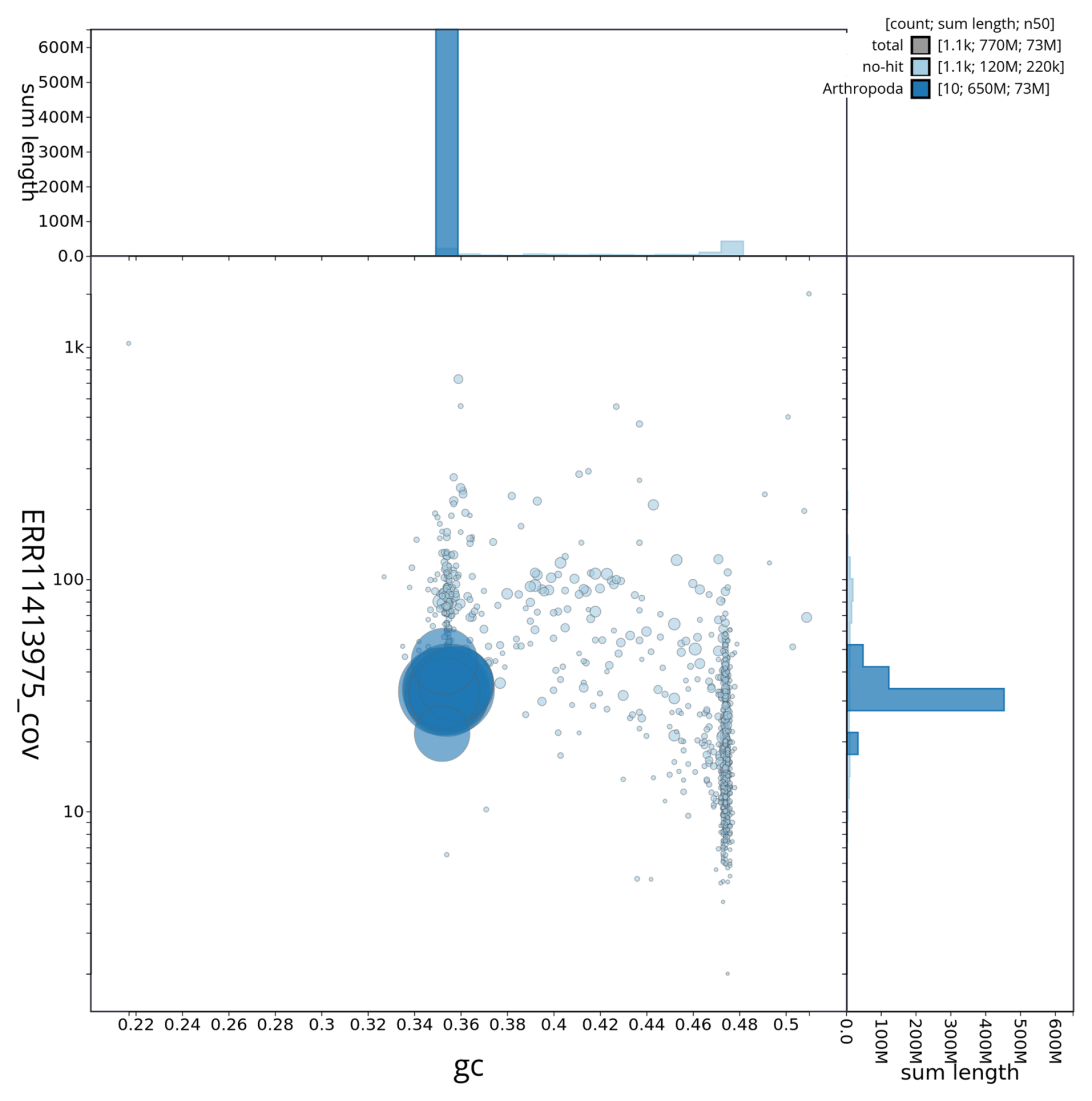
Genome assembly of *Myrrha octodecimguttata*, icMyrOcto1.1: BlobToolKit GC-coverage plot. BlobToolKit GC-coverage plot showing sequence coverage (vertical axis) and GC content (horizontal axis). The circles represent scaffolds, with the size proportional to scaffold length and the colour representing phylum membership. The histograms along the axes display the total length of sequences distributed across different levels of coverage and GC content. An interactive version of this figure is available at https://blobtoolkit.genomehubs.org/view/icMyrOcto1_1/dataset/icMyrOcto1_1/blob.

**Figure 4.**
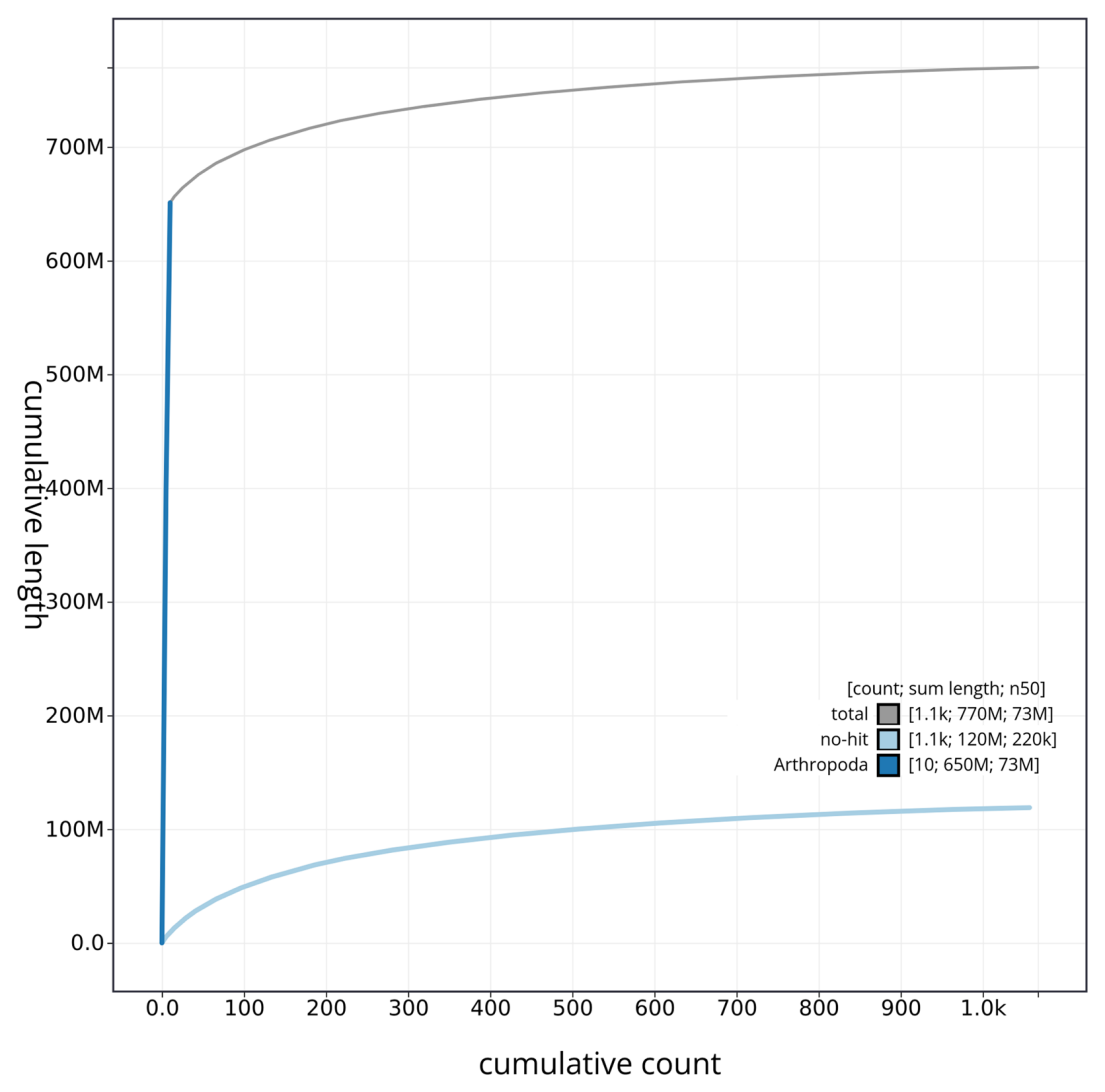
Genome assembly of *Myrrha octodecimguttata* icMyrOcto1.1: BlobToolKit cumulative sequence plot. The grey line shows cumulative length for all sequences. Coloured lines show cumulative lengths of sequences assigned to each phylum using the buscogenes taxrule. An interactive version of this figure is available at https://blobtoolkit.genomehubs.org/view/icMyrOcto1_1/dataset/icMyrOcto1_1/cumulative.

The snail plot in Figure 2 provides a summary of the assembly statistics, indicating the distribution of scaffold lengths and other assembly metrics. Figure 3 shows the distribution of scaffolds by GC proportion and coverage. Figure 4 presents a cumulative assembly plot, with separate curves representing different scaffold subsets assigned to various phyla, illustrating the completeness of the assembly.

**Figure 5.**
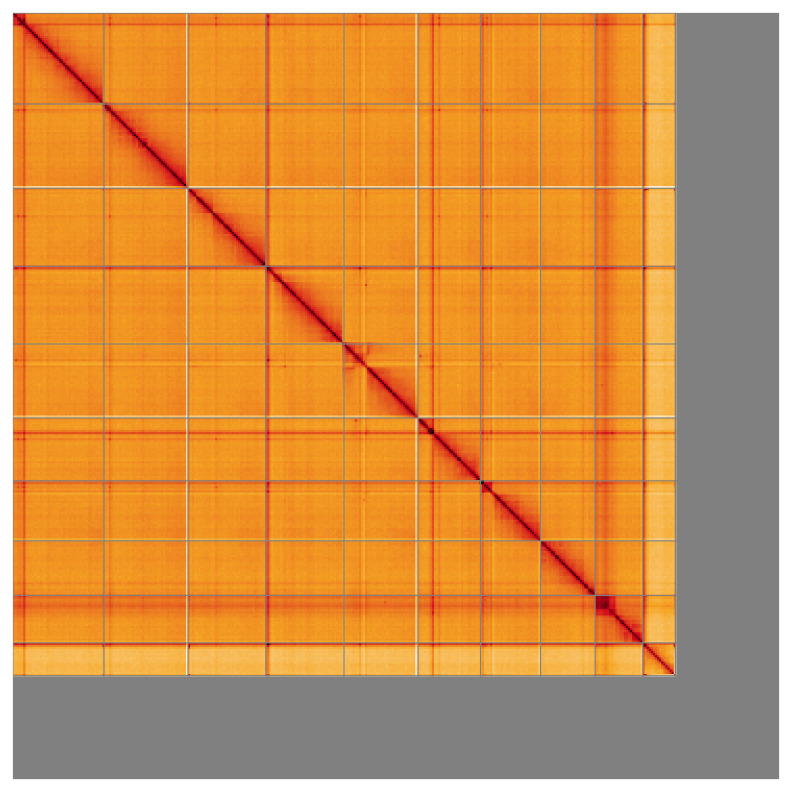
Genome assembly of *Myrrha octodecimguttata* icMyrOcto1.1: Hi-C contact map of the icMyrOcto1.1 assembly, visualised using HiGlass. Chromosomes are shown in order of size from left to right and top to bottom. An interactive version of this figure may be viewed at https://genome-note-higlass.tol.sanger.ac.uk/l/?d=Sh7ZoZtCRrCgk_qUjCUjOA.

Most of the assembly sequence (84.54%) was assigned to 10 chromosomal-level scaffolds, representing 9 autosomes and the X sex chromosome. These chromosome-level scaffolds, confirmed by the Hi-C data, are named in order of size ( Figure 5; Table 3). During manual curation it was noted that Chromosome X was assigned based on read coverage statistics. No Y could be identified, and the species may be XO male.

**Table 3.** Chromosomal pseudomolecules in the genome assembly of *Myrrha octodecimguttata*, icMyrOcto1.

INSDC accession	Name	Length (Mb)	GC%
OY294056.1	1	89.46	35.5
OY294057.1	2	82.69	35.0
OY294058.1	3	76.63	35.5
OY294059.1	4	76.43	35.5
OY294060.1	5	72.74	35.5
OY294061.1	6	61.6	36.0
OY294062.1	7	58.94	36.0
OY294063.1	8	53.6	35.5
OY294064.1	9	46.72	35.5
OY294065.1	X	32.15	35.0
OY294066.1	MT	0.02	22.0

While not fully phased, the assembly deposited is of one haplotype. Contigs corresponding to the second haplotype have also been deposited. The mitochondrial genome was also assembled and can be found as a contig within the multifasta file of the genome submission, and as a separate fasta file with accession OY294066.1.

The final assembly has a Quality Value (QV) of 64.0 and *k*-mer completeness of 98.08% for the combined assemblies. BUSCO (v5.4.3) analysis using the endopterygota_odb10 reference set ( *n* = 2,124) indicated a completeness score of 97.8% (single = 96.0%, duplicated = 1.8%). The assembly achieves the EBP reference standard of 6.7.64.0. Other quality metrics are given in Table 2. 

### Genome annotation report

The *Myrrha octodecimguttata* genome assembly (GCA_958510865.1) was annotated at the European Bioinformatics Institute (EBI) on Ensembl Rapid Release. The resulting annotation includes 22,665 transcribed mRNAs from 22,423 protein-coding genes ( Table 2; https://rapid.ensembl.org/Myrrha_octodecimguttata_GCA_958510865.1/Info/Index). The average transcript length is 6,417.66, with an average of 3.98 exons per transcript.

## Methods

### Sample acquisition and DNA barcoding

An adult male specimen of *Myrrha octodecimguttata* (specimen ID NHMUK014440586, ToLID icMyrOcto1) was collected from Brompton Cemetery, Kensington and Chelsea, England, United Kingdom (latitude 51.48, longitude -0.19) on 2022-03-18. The specimen was collected and identified by Maxwell Barclay (Natural History Museum) and preserved by dry freezing at –80 °C.

The initial identification was verified by an additional DNA barcoding process according to the framework developed by Twyford *et al.* (2024). A small sample was dissected from the specimens and stored in ethanol, while the remaining parts were shipped on dry ice to the Wellcome Sanger Institute (WSI). The tissue was lysed, the COI marker region was amplified by PCR, and amplicons were sequenced and compared to the BOLD database, confirming the species identification ( Crowley *et al.*, 2023). Following whole genome sequence generation, the relevant DNA barcode region was also used alongside the initial barcoding data for sample tracking at the WSI ( Twyford *et al.*, 2024). The standard operating procedures for Darwin Tree of Life barcoding have been deposited on protocols.io ( Beasley *et al.*, 2023).

### Nucleic acid extraction

The workflow for high molecular weight (HMW) DNA extraction at the Wellcome Sanger Institute (WSI) Tree of Life Core Laboratory includes a sequence of procedures: sample preparation and homogenisation, DNA extraction, fragmentation and purification. Detailed protocols are available on protocols.io ( Denton *et al.*, 2023b). The icMyrOcto1 sample was prepared for DNA extraction by weighing and dissecting it on dry ice ( Jay *et al.*, 2023). Tissue from the whole organism was homogenised using a PowerMasher II tissue disruptor ( Denton *et al.*, 2023a).

HMW DNA was extracted in the WSI Scientific Operations core using the Automated MagAttract v2 protocol ( Oatley *et al.*, 2023). The DNA was sheared into an average fragment size of 12–20 kb in a Megaruptor 3 system ( Bates *et al.*, 2023). Sheared DNA was purified by solid-phase reversible immobilisation, using AMPure PB beads to eliminate shorter fragments and concentrate the DNA ( Strickland *et al.*, 2023). The concentration of the sheared and purified DNA was assessed using a Nanodrop spectrophotometer and Qubit Fluorometer using the Qubit dsDNA High Sensitivity Assay kit. Fragment size distribution was evaluated by running the sample on the FemtoPulse system.

### Hi-C preparation

Tissue from the icMyrOcto1 sample was processed at the WSI Scientific Operations core, using the Arima-HiC v2 kit. Tissue (stored at –80 °C) was fixed, and the DNA crosslinked using a TC buffer with 22% formaldehyde. After crosslinking, the tissue was homogenised using the Diagnocine Power Masher-II and BioMasher-II tubes and pestles. Following the kit manufacturer's instructions, crosslinked DNA was digested using a restriction enzyme master mix. The 5’-overhangs were then filled in and labelled with biotinylated nucleotides and proximally ligated. An overnight incubation was carried out for enzymes to digest remaining proteins and for crosslinks to reverse. A clean up was performed with SPRIselect beads prior to library preparation.

### Library preparation and sequencing

Library preparation and sequencing were performed at the WSI Scientific Operations core. Pacific Biosciences HiFi circular consensus DNA sequencing libraries were prepared using the PacBio Express Template Preparation Kit v2.0 (Pacific Biosciences, California, USA) as per the manufacturer's instructions. The kit includes the reagents required for removal of single-strand overhangs, DNA damage repair, end repair/A-tailing, adapter ligation, and nuclease treatment. Library preparation also included a library purification step using AMPure PB beads (Pacific Biosciences, California, USA) and size selection step to remove templates shorter than 3 kb using AMPure PB modified SPRI. DNA concentration was quantified using the Qubit Fluorometer v2.0 and Qubit HS Assay Kit and the final library fragment size analysis was carried out using the Agilent Femto Pulse Automated Pulsed Field CE Instrument and gDNA 165kb gDNA and 55kb BAC analysis kit. Samples were sequenced using the Sequel IIe system (Pacific Biosciences, California, USA). The concentration of the library loaded onto the Sequel IIe was in the range 40–135 pM. The SMRT link software, a PacBio web-based end-to-end workflow manager, was used to set-up and monitor the run, as well as perform primary and secondary analysis of the data upon completion.

For Hi-C library preparation, DNA was fragmented to a size of 400 to 600 bp using a Covaris E220 sonicator. The DNA was then enriched, barcoded, and amplified using the NEBNext Ultra II DNA Library Prep Kit following manufacturers’ instructions. The Hi-C sequencing was performed using paired-end sequencing with a read length of 150 bp on an Illumina NovaSeq 6000 instrument.

### Genome assembly, curation and evaluation

***Assembly***

The HiFi reads were first assembled using Hifiasm ( Cheng *et al.*, 2021) with the --primary option. Haplotypic duplications were identified and removed using purge_dups ( Guan *et al.*, 2020). The Hi-C reads were mapped to the primary contigs using bwa-mem2 ( Vasimuddin *et al.*, 2019). The contigs were further scaffolded using the provided Hi-C data ( Rao *et al.*, 2014) in YaHS ( Zhou *et al.*, 2023) using the --break option for handling potential misassemblies. The scaffolded assemblies were evaluated using Gfastats ( Formenti *et al.*, 2022), BUSCO ( Manni *et al.*, 2021) and MERQURY.FK ( Rhie *et al.*, 2020).

The mitochondrial genome was assembled using MitoHiFi ( Uliano-Silva *et al.*, 2023), which runs MitoFinder ( Allio *et al.*, 2020) and uses these annotations to select the final mitochondrial contig and to ensure the general quality of the sequence.

***Assembly curation***

The assembly was decontaminated using the Assembly Screen for Cobionts and Contaminants (ASCC) pipeline (article in preparation). Manual curation was primarily conducted using PretextView ( Harry, 2022), with additional insights provided by JBrowse2 ( Diesh *et al.*, 2023) and HiGlass ( Kerpedjiev *et al.*, 2018). Scaffolds were visually inspected and corrected as described by Howe *et al.* (2021). Any identified contamination, missed joins, and mis-joins were corrected, and duplicate sequences were tagged and removed. The sex chromosomes were assigned based on read coverage statistics. The curation process is documented at https://gitlab.com/wtsi-grit/rapid-curation (article in preparation).

***Evaluation of the final assembly***

A Hi-C map for the final assembly was produced using bwa-mem2 ( Vasimuddin *et al.*, 2019) in the Cooler file format ( Abdennur & Mirny, 2020). To assess the assembly metrics, the *k*-mer completeness and QV consensus quality values were calculated in Merqury ( Rhie *et al.*, 2020). This work was done using Nextflow ( Di Tommaso *et al.*, 2017) DSL2 pipelines “sanger-tol/readmapping” ( Surana *et al.*, 2023a) and “sanger-tol/genomenote” ( Surana *et al.*, 2023b). The genome evaluation pipelines were developed using nf-core tooling ( Ewels *et al.*, 2020) and MultiQC ( Ewels *et al.*, 2016), relying on the Conda package manager, the Bioconda initiative ( Grüning *et al.*, 2018), the Biocontainers infrastructure ( da Veiga Leprevost *et al.*, 2017), as well as the Docker ( Merkel, 2014) and Singularity ( Kurtzer *et al.*, 2017) containerisation solutions. The genome was also analysed within the BlobToolKit environment ( Challis *et al.*, 2020) and BUSCO scores ( Manni *et al.*, 2021) were calculated.

Table 4 contains a list of relevant software tool versions and sources.

**Table 4.** Software tools: versions and sources.

Software tool	Version	Source
BlobToolKit	4.2.1	https:////github.com/blobtoolkit/blobtoolkit
BUSCO	5.3.2	https:////gitlab.com/ezlab/busco
bwa-mem2	2.2.1	https:////github.com/bwa-mem2/bwa-mem2
Cooler	0.8.11	https:////github.com/open2c/cooler
FastK	427104ea91c78c3b8b8b49f1a7d6bbeaa869ba1c	https:////github.com/thegenemyers/FASTK
Gfastats	1.3.6	https:////github.com/vgl-hub/gfastats
Hifiasm	0.16.1-r375	https:////github.com/chhylp123/hifiasm
HiGlass	1.11.6	https:////github.com/higlass/higlass
HiGlass	44086069ee7d4d3f6f3f0012569789ec138f42b84 aa44357826c0b6753eb28de	https:////github.com/higlass/higlass
Merqury	MerquryFK	https:////github.com/thegenemyers/MERQURY.FK
Merqury.FK	d00d98157618f4e8d1a9190026b19b471055b22e	https:////github.com/thegenemyers/MERQURY.FK
MitoHiFi	2	https:////github.com/marcelauliano/MitoHiFi
Nextflow	23.04.0-5857	https:////github.com/nextflow-io/nextflow
PretextView	0.2	https:////github.com/wtsi-hpag/PretextView
purge_dups	1.2.3	https:////github.com/dfguan/purge_dups
sanger-tol/ascc	-	https:////github.com/sanger-tol/ascc
sanger-tol/genomenote	v1.0	https:////github.com/sanger-tol/genomenote
sanger-tol/readmapping	1.1.0	https:////github.com/sanger-tol/readmapping/tree/1.1.0
Singularity	3.9.0	https:////github.com/sylabs/singularity
YaHS	yahs-1.1.91eebc2	https:////github.com/c-zhou/yahs

### Genome annotation

The BRAKER2 pipeline ( Brůna *et al.*, 2021) was used in the default protein mode to generate annotation for the *Myrrha octodecimguttata* assembly (GCA_958510865.1) in Ensembl Rapid Release at the EBI.

### Wellcome Sanger Institute – Legal and Governance

The materials that have contributed to this genome note have been supplied by a Darwin Tree of Life Partner. The submission of materials by a Darwin Tree of Life Partner is subject to the **‘Darwin Tree of Life Project Sampling Code of Practice’**, which can be found in full on the Darwin Tree of Life website here. By agreeing with and signing up to the Sampling Code of Practice, the Darwin Tree of Life Partner agrees they will meet the legal and ethical requirements and standards set out within this document in respect of all samples acquired for, and supplied to, the Darwin Tree of Life Project.

Further, the Wellcome Sanger Institute employs a process whereby due diligence is carried out proportionate to the nature of the materials themselves, and the circumstances under which they have been/are to be collected and provided for use. The purpose of this is to address and mitigate any potential legal and/or ethical implications of receipt and use of the materials as part of the research project, and to ensure that in doing so we align with best practice wherever possible. The overarching areas of consideration are:

•    Ethical review of provenance and sourcing of the material

•    Legality of collection, transfer and use (national and international)

Each transfer of samples is further undertaken according to a Research Collaboration Agreement or Material Transfer Agreement entered into by the Darwin Tree of Life Partner, Genome Research Limited (operating as the Wellcome Sanger Institute), and in some circumstances other Darwin Tree of Life collaborators.

## Data Availability

European Nucleotide Archive: *Myrrha octodecimguttata*. Accession number PRJEB61846; https://identifiers.org/ena.embl/PRJEB61846. The genome sequence is released openly for reuse. The *Myrrha octodecimguttata* genome sequencing initiative is part of the Darwin Tree of Life (DToL) project. All raw sequence data and the assembly have been deposited in INSDC databases. Raw data and assembly accession identifiers are reported in Table 1 and Table 2. Metadata for specimens, BOLD barcode results, spectra estimates, sequencing runs, contaminants and pre-curation assembly statistics are given at https://links.tol.sanger.ac.uk/species/703265.

## Author information

Members of the Natural History Museum Genome Acquisition Lab are listed here: https://doi.org/10.5281/zenodo.12159242.

Members of the Darwin Tree of Life Barcoding collective are listed here: https://doi.org/10.5281/zenodo.12158331.

Members of the Wellcome Sanger Institute Tree of Life Management, Samples and Laboratory team are listed here: https://doi.org/10.5281/zenodo.12162482.

Members of Wellcome Sanger Institute Scientific Operations: Sequencing Operations are listed here: https://doi.org/10.5281/zenodo.12165051.

Members of the Wellcome Sanger Institute Tree of Life Core Informatics team are listed here: https://doi.org/10.5281/zenodo.12160324.

Members of the Tree of Life Core Informatics collective are listed here: https://doi.org/10.5281/zenodo.12205391.

Members of the Darwin Tree of Life Consortium are listed here: https://doi.org/10.5281/zenodo.4783558.
