# Supplementary material for: The genome sequence of 18-spot Ladybird, Myrrha octodecimguttata (Linnaeus, 1758)
Source: Wellcome Open Res. 2025 Jan 14;10:7. [Version 1] doi: 10.12688/wellcomeopenres.23455.1 (PMC12461194; doi:10.12688/wellcomeopenres.23455.1)
Supplement: Peer review history

# Reviewer report: Reviewer response for version 1

**DOI:** 10.21956/wellcomeopenres.25865.r132539

**Published:** 2025-09-24

**Authors:** Marta Coronado-Zamora

**Affiliations:** 1 Autonomous University of Barcelona Department of Genetics and Microbiology (Ringgold ID: 196650), Bellaterra, Catalonia, Spain; 2 Institute of Evolutionary Biology, Barcelona, Spain

This data note describes a high-quality assembly of the 18-spot ladybird, using the most advanced sequencing and assembly methods. The genome data are available at NCBI and data is accessible as checked in September 2025.

 I only have some minor comments:

 - Remove labels a), b) and c) from Figure 1 if they are not used. 

 - Table 1: present the read count in a non-scientific format would make it easy to read.

 - Table 4 has extra "//" symbols in the source columns.

 - Software missing from table 4 and mentioned in the text: JBrowse2 and BRAKER2.

 - If known, introducing the sex determination of this specifies could be useful.

Are sufficient details of methods and materials provided to allow replication by others?

Yes

Is the rationale for creating the dataset(s) clearly described?

Yes

Are the datasets clearly presented in a useable and accessible format?

Yes

Are the protocols appropriate and is the work technically sound?

Yes

Reviewer Expertise:

bioinformatics, population genetics, genomics

I confirm that I have read this submission and believe that I have an appropriate level of expertise to confirm that it is of an acceptable scientific standard.

# Reviewer report: Reviewer response for version 1

**DOI:** 10.21956/wellcomeopenres.25865.r118108

**Published:** 2025-02-25

**Authors:** Mariana A Cherman

**Affiliations:** 1 Universidade Federal do Parana, Curitiba, State of Paraná, Brazil

This paper presents the complete genome dataset of the 18-spotted ladybird, Myrrha octodecimguttata.

 There are few specimens with sequenced genomes, which is why it is important to index it.

 Just some minor corrections, when a sentence is initializing with the name of the species M. octodecimguttata, the generic name should not be abbreviated, i.e.: Myrrha instead of M.

Are sufficient details of methods and materials provided to allow replication by others?

Yes

Is the rationale for creating the dataset(s) clearly described?

Yes

Are the datasets clearly presented in a useable and accessible format?

Yes

Are the protocols appropriate and is the work technically sound?

Yes

Reviewer Expertise:

entomology, taxonomist

I confirm that I have read this submission and believe that I have an appropriate level of expertise to confirm that it is of an acceptable scientific standard.
